# M. Belhomme on Political and Epidemic Insanity

**Published:** 1848-07-01

**Authors:** 


Art. IV.—*L'effet d'Emotions politiques, dans la production de la Folie.* Par M. Belhomme. (Read at the sitting of the Académie de Médecine, 1848.)

When the Roman satirist, with infinite grace and humour, discussed with Damisippus the paradox of the Stoic philosophy, that *omnes propemodùm homines insanire,* he had in view the school and sect of Chrysippus, which deemed every man *mad* whom vicious folly or wilful ignorance drove blindly forward. Under this sweeping definition, what kings, what conquerors, heroes,—nay, what whole tribes and nations are included? And yet, when we consider the trivial agents that act upon man's mind to destroy the balance, the influence of unworthy impulses to give it an unhealthy bias, and make it run riot in folly and extravagance, we are tempted almost to doubt whether *sanity* is a term ever fit to be applied to the human intellect. *Non omnes propemodùm sed omnes homines insanire.* Look to the page of history for a confirmation.

The pride of man induces him to believe that the whole arcana of nature are revealed to him and him alone. That that Intelligence with which he is endowed enables him to penetrate the secret springs of action, and lay bare the laws of the Eternal. Alas! the boasted light of Reason, how feeble you become when you attempt to lead your master beyond the confines of the physical! Your dim and flickering ray serves but to make more visible the Cimmerian darkness, and to fill the void with wild, imaginative spectres! Yet would man's prerogative be employed most usefully, were its powers equal to the task of grappling with the world unseen. Powers are revealed to him daily through the medium of his senses, of which he can take note and cognizance; but assuredly, also, there are powers, mighty and mysterious powers! beyond the appreciation of sense, which exert no inconsiderable influence on his present and his future destiny.

One of these powers is *sympathy.* A strange, mysterious instinct, that binds man to man with a chain, not less secure because its links are hidden. It accounts for the union of mankind into a social whole, and while it is the basis of society, places self-independence in a very doubtful light. From it originate the best emotions of the soul. Compassion, pity, mercy, and benevolence, are among its genial offspring. But still it embraces with equal force, reason and folly, good and evil; and thus, by its indiscriminating influence, it lessens the beauty of virtue as well as the deformity of vice.

Closely allied to sympathy, and serving often as its exponent, we have *imitation.* Present with the infant in the cradle, and aiding his earliest intellectual efforts, it bears no mean or trifling sway throughout the whole of life. In moderation and in health, it doubtless teems with rich and goodly fruit; but in excess or in disease, abounds with evils of great and fearful magnitude. When exerted in its highest degree, it becomes a mental bondage. Then it is that the sensible impression of a nervous malady fetters the will, and produces a condition like that of fascination. The mind is enchained by the serpent's eye —the spell is irresistible. Reason is overthrown, the will opposed and overpowered, and the soul given over to a strange and demoniacal influence. No skill of the physician, no menace of the magistrate, can stay the foaming torrent—onwards, headlong it rushes, and the natural impulse asserts and gains its supremacy—

> "Naturam expellas furcâ,
> Tamen usque recurret."

To morbid sympathy, and that demon of imitation to which we have alluded, the diffusion of violent excitements is due, especially those of a religious or political character, which have so powerfully agitated the nations of ancient and modern times. These may, by habitual compliance, pass into a total loss of power over the will and an actual disease of the mind. Witness the immense increase in the number of the insane that accompanies every great revolution. The secret springs of action lie hidden in the inmost recesses of the soul: but truly the instinct of imitation is often a key to man's most triumphant efforts for good or evil. Sympathy lays bare his weakest and most vulnerable side.

But independently of political and religious excitement, which may seem to be more or less based on reason and intelligence, we have another class of affections which afford a deep insight into the workings of the human mind in its social relationship. They are closely connected with life in the aggregate, and are propagated on the beams of light—on the wings of thought. They, as has been well remarked, convulse the mind by the excitement of the senses, and wonderfully affect the nerves, the media of its will and of its feelings. The whole world is full of examples of this afflicting state of turmoil, which, when the mind is carried away by the force of a sensual impression that destroys its freedom, is irresistibly propagated by imitation. Those who are thus infected do not spare even their own lives, but as a hunted flock of sheep will follow their leader and rush over a precipice, so will whole hosts of enthusiasts, deluded by their infatuation, hurry on to a self-inflicted death. Such has ever been the case, from the days of the Milesian virgins to the modern associations for self-destruction.

The intimate union of the mind and body is shown by their mutual reaction on each other. When one is disturbed, the other is invariably more or less out of order. The body being mortal is subject to disease. The mind also has its peculiar ailments. Some of these corporeal affections are single, isolated. Others again are endemic, epidemic. Strange that in this respect the analogy between mind and matter still holds good! Psychical affections are also frequently epidemic, and what is more, are powerfully contagious. Sympathy and imitation appear to be the subtle poisons that convey the infection from one person to another, and those who are of a nervous, excitable temperament, are most subject to their pernicious influence. They are enemies that enter the brain through the senses and take the reason prisoner. Those who are bitten by the mania,

> "Play such fantastic tricks before high heaven,

> As make the angels weep."

It is this contagious malady of the nervous system that we propose to examine in this paper. We use the word *contagious* for want of a better, although we are well aware of the objections that may be urged against it. Contagion can, in a psychical sense, signify nothing but pathological sympathy. The materials, unfortunately, are so abundant, so replete is the book of history with their details, that our only difficulty is in making a selection. It is *l'embarras de richesses* that troubles us. Passing notices are to be found scattered in the writings of both ancient and modern authors, but by far the richest store is that contained in Hecker's "Epidemics of the Middle Ages,"—a valuable and philosophic production.

Familiar illustrations of the effects of sympathy and the contagious nature of imitation, are witnessed in the acts of sneezing, coughing, &c. If one person coughs in a church, others will do so likewise. The act of yawning, again, is irresistibly infectious. There is a well-known story of a learned professor, in Modern Athens, who had suffered from a dislocation of the jaw, which displacement always recurred whenever he opened his mouth too wide. His more idle pupils used to take advantage of this circumstance, whenever they thought his lecture was too prosy. One sitting in front of him would pretend to yawn. The professor could not resist the fascination; he yawned too, and consequently the bone was luxated. In the confusion consequent upon this accident the roguish students made their escape from the lecture room.

The contagious nature of laughter must have been noticed by every one. People frequently are half dead with merriment, who can assign no reason for the impulse. They do not know what the joke is. They laugh because others laugh. Sometimes the propensity to cachinnation is produced through the sense of sight, as when whole audiences have roared at the first glance at Reeves, Liston, or Charles Mathews. More frequently it is through the portals of the ears that the poison is distilled. A lady states that she was lying ill in bed the other day, in a chamber next to one in which a merry company was assembled. Although she could not catch a word of the conversation, she, in spite of herself, laughed heartily every time her neighbours laughed, although the act gave her great agony. The emotion observed in mobs and other great assemblies is of the same nature. The most trifling accident will turn the tide one way or the other. Hence all observers have noticed the fickleness of the multitude, and how easily it is swayed to good or evil.

These are familiar illustrations of mental and emotional contagion. We will now proceed to investigate the subject in its historical and moral bearings.

Caspar, Esquirol, and Pinel, have established the important fact, that moral predominate over physical causes in the production of insanity among civilized people. The proportion is as nearly two to one. And what greater proof of this can we desire than the reigning absurdities and extravagances of each country and each epoch? The political crises which strike at the root of social order, the remarkable events which stand out in bold relief and give a feature to the age, belong not solely to history. They come within the department of medicine, which by their means could form a striking picture of human vicissitudes. Thus, when a political catastrophe has occasioned the ruin or death of a great number of persons, the apparent victims are not the only indications of the disaster. The blow is felt at a much greater distance. It reacts on the minds of a multitude of silly persons, who are only too ready for the infection. In fact, there always exists on the surface of society a large floating mass of individuals, devoted by their organization to mental alienation. Weak minded, wild, and conceited, frequently the issue of eccentric parents, their brain receives, like softened wax, every impression that is offered; and their reason, perverted by a vicious education and defective organization, not being able to withstand the shock, is permanently overthrown. These people are easily recognised by their manners, their gestures, and conversation; their excessive gaiety without motive; the rapid succession of their ideas; the facility with which they form and as readily abandon a hundred wild projects. Irritability of disposition, wildness of the eyes, eccentricity of conduct, certain nervous actions or movements, looseness and feebleness of ideas, want of force and connexion in ratiocination, are all pathognomonic signs which presage their future destiny. In fact, it is possible to foretel by the physiognomy, and we mean by that the whole exterior, that such and such a man will become insane, as we should say of another that the probability is he will die of apoplexy or consumption.

The influence of predominating ideas and example upon these individuals is really astonishing. Thus it was in ancient Greece, when religious festivals, and especially the celebration of the mysteries of Bacchus upon the heights of Parnassus, gave rise to the strangest disorders of the intellect. Then were seen whole hosts of frantic women, half naked, and with dishevelled hair, rushing like maniacs through towns and provinces, giving utterance to savage and demoniac yells. The slightest spark would enkindle the blaze. Some of them, suddenly seized with delirium, fancied themselves gifted with divine inspiration, and infected their companions with the hallucination. The excesses of the Athenian matrons will not surprise those who know how easy it is to excite the lively imagination and ardent temperament of the female sex. How far they were influenced by wine is another question.

The course and progress of the epidemic is well shown by the fury of the mother and aunt of Pentheus, in Ovid's Metamorphoses—

"For now, through prostrate Greece, young Bacchus rode,
While howling matrons celebrate the god.
All ranks and sexes to his orgies ran
To mingle in the pomps, and fill the train."

The Theban king determines to see with his own eyes the mystic ceremonies. He approaches—

"A spacious circuit on the hill there stood,
Level and wide, and skirted round with wood;
There the rash Pentheus, with unhallowed eyes,
The howling dames and mystic orgies spies.
* * * * * * *
In vain does Pentheus to his mother sue,
And the raw bleeding stumps present to view.

His mother howled, and heedless of his prayer,
Her trembling hand she twisted in his hair.
'And this,' she cried, 'shall be Agave's share.'
When from his neck his struggling head she tore,
And in her hands the ghastly visage bore,
With pleasure all the hideous trunk survey,
Then pulled and tore the mangled limbs away,
As, starting in the pangs of death it lay.
With such a sudden death lay Pentheus slain,
And in a thousand pieces strewed the plain."

This story of the Theban king having been torn in pieces by his nearest relatives, does not seem wholly fabulous, when we consider the maniacal fury of those suffering from epidemic infatuation.

In the latter days of the Roman republic, under the emperors, epidemic suicide was frequent in consequence of the continual proscriptions, executions, and tyrannical decrees. Through fear and despair, immense numbers of senators and other distinguished individuals put a period to their existence. For self-murder is a frequent resource among people who are depressed, and are not sustained by religious faith. Death then appears a sure refuge from trouble. The irruption of the barbarians, by increasing the terror, desolation, and ruin, in all parts of the empire, necessarily increased the tendency to mental alienation. The persecution of the early Christians had the same effect. What other result could be expected from the blazing faggot, the rack, and combats with ferocious animals? Such spectacles either heat the imagination to the highest degree, or chill it by terror; and such are the fittest states for the access of lunacy. Imitation, that true moral contagion, completed the work, and rendered the disorder epidemic.

In following the chronological order of periods, we come to the grand era of the middle ages, which furnishes astonishing examples of imitative mania, in all its variety, in all its intensity. But here, before entering into a description of this strange moral malady, let us cast our eye at the state of the public mind and the situation of Europe at that epoch.

Perpetual foreign wars and intestine commotions, irruptions of barbarians, priestly intervention, profound ignorance of both high and low, and the love of the marvellous which is its natural consequence, had developed to an extraordinary degree the passion for arms, religious enthusiasm, and superstitious faith in supernatural visitation. Alchemy and judicial astronomy had contributed to turn the heads of men. Thus, the moment the voice of Peter the hermit was heard, a universal delirium seized upon the mind. Old and young, men, women, and children, followed in the footsteps of the monk. It appeared as if the western world had been heaved from its foundations, in order to be precipitated upon the east. Can this infatuation be accounted for in any other way than by imitation and morbid sympathy? Abundant instances were then afforded of disturbance of the intellect. The annals of the crusades are filled with apparitions of angels and of saints—of divine revelations and fabulous exploits. Hallucinations of the sight and hearing were also extremely common. In the first two crusades the type of aberration was of a religious and warlike character.

In the third, the Franks changed the epic character of their warrior manners for that of the romantic. Hence the reign of troubadours and chivalric knights, who, directing the imagination of the public towards love and glory, gave another tint to the mental alienation. Erotomania, nymphomania, several varieties of hysteria, and the mania for warlike exploits, were the distinctive features of that epoch. Roland and King Arthur were types of the period. It would be wrong, therefore, to refer their strange and eccentric actions exclusively to madness, when they may, in a great measure, be traced to the prevailing religion, manners and customs, laws, and superstitions of the times. You may define a lunatic to be one who sets out from a false, exaggerated, fantastic, or imaginary principle, and reasons upon it as if it were true.

It was in the eleventh century, so celebrated for the first crusade, that the earliest signs of the epidemic Dancing Mania made their appearance. During its access, the patients fought with each other like madmen, leapt with savage gestures to an enormous height, and wounded themselves and others. They fancied they heard strange sounds and voices in their ears. At the slightest breath of music they abandoned themselves to convulsive dancing, till they were exhausted by fatigue. These dangerous maniacs were regarded as forming part of the legion of Satan. Possibly a similar malady was prevalent at the time of the foundation of Christianity. Persons were then said to be tormented by devils, and were cured only by miracle.

If we regard the events succeeding each other from the twelfth to the close of the fourteenth century, we shall perceive ample physical causes to account for the increase of the dancing mania. If the body would ever react on the mind, this would be the time to perceive its agency. Nearly all the countries of Europe were ravaged by horrible epidemics. The measles and small-pox took off tens of thousands. St. Anthony's fire was the terror of town and country. The domestic hearth supplied innumerable victims to the leprosy. Finally, the plague, or as it was called, the *Black Death,* made its appearance (1350) and swept away millions of persons. The consternation and misery produced by this succession of evils kept the mind in a continual tension, and exasperated the symptoms of the moral ailment. Everything tended to a disturbance of the nervous system, and we shall see to what a fearful extent this was carried.

As our inquiry is much facilitated by the labours of the learned Hecker, we cannot do better than let him speak for himself. He says, at the commencement of his description of that variety of the dancing mania called the "St. John's Dance":—

"The effects of the black death had not yet subsided, and the graves of millions of its victims were scarcely closed, when a strange delusion arose in Germany, which took possession of the minds of men, and in spite of the divinity of our nature, hurried away body and soul into the magic circle of hellish superstition. It was a convulsion which in the most extraordinary manner infuriated the human frame, and excited the astonishment of contemporaries for more than two centuries, since which time it has never re-appeared. It was called the dance of St. John, or of St. Vitus, on account of the Bacchantic leaps by which it was characterized, and which gave to those affected, whilst performing their wild dance and screaming and foaming with fury, all the appearance of persons possessed. It did not remain confined to particular localities, but was propagated by the sight of the sufferers, like a demoniacal epidemic, over the whole of Germany and the neighbouring countries to the north-west, which were already prepared for its reception by the prevailing opinions of the times.

"So early as the year 1374, assemblages of men and women were seen at Aix-la-Chapelle, who had come out of Germany, and who, united by one common delusion, exhibited to the public, both in the streets and in the churches, the following strange spectacle:—They formed circles, hand in hand, and appearing to have lost all control over their senses, continued dancing, regardless of the by-standers, for hours together, in wild delirium, until at length they fell to the ground, in a state of exhaustion. They then complained of extreme oppression, and groaned as if in the agonies of death, until they were swathed in cloths, bound tightly round their waists, upon which they again recovered, and remained free from complaint until the next attack. This practice of swathing was resorted to on account of the tympany which followed these spasmodic ravings; but the by-standers frequently relieved patients in a less artificial manner, by thumping and trampling upon the parts affected. While dancing, they neither saw nor heard, being insensible to external impressions through the senses, but were haunted by visions, their fancies conjuring up spirits whose names they shrieked out; and some of them afterwards asserted that they felt as if they had been immersed in a stream of blood, which obliged them to leap so high; and thus, during the paroxysm, saw the heavens open and the Saviour enthroned with the Virgin Mary, according as the religious notions of the age were strangely and variously reflected in their imagination. When the disease was completely developed, the attack commenced with epileptic convulsions. Those affected fell to the ground senseless, panting and labouring for breath. They foamed at the mouth, and suddenly springing up, began their dance amidst strange contortions."

In a few months this demoniacal disease spread from Aix-la-Chapelle over the neighbouring Netherlands. In Liege, Utrecht, Tongres, and many other towns of Belgium, the dancers appeared with garlands in their hair, and their waists girt with cloths, that they might, as soon as the paroxysm was over, receive immediate relief on the attack of the tympany. This bandage was, by the insertion of a stick, easily twisted tight; many, however, obtained more relief from kicks and blows, which they found numbers of persons ready to administer; for wherever the dancers appeared, the people assembled in crowds to gratify their curiosity by the frightful spectacle. Some objects appeared to have great effect in increasing the excitement. Thus they were greatly irritated by the sight of red colours, the influence of which on the disordered nerves might lead us to imagine an extraordinary accordance between this spasmodic malady and the condition of infuriated animals. There were some of them who were unable to endure the sight of persons weeping, and others who manifested such a morbid dislike to the pointed shoes which had come into fashion immediately after the great mortality, that the authorities were obliged to issue an ordinance against the manufacture of any but those with square toes.

The number of these fanatics must have been great, for they frequently assembled in multitudes, and menaced the magistrates and priests with destruction. The mass of the people was intimidated, and had no doubt of the demoniacal origin of the disease. Everywhere horror and astonishment were created. Processions were everywhere instituted on their account, while masses were said, and hymns were sung, to dispel the evil spirit. Exorcisms were also a favourite remedy with the priests.

The frenzy broke out at Cologne a few months after its appearance at Aix, and the number of those possessed amounted to more than five hundred. In Metz, the streets were said to have been filled with eleven hundred dancers. Peasants left their ploughs, mechanics their workshops, housewives their domestic duties, to join the wild revels; and this rich commercial city became the scene of the most ruinous disorder. Girls and boys quitted their parents, and servants their masters, to amuse themselves at the dances of those possessed, and greedily imbibed the poison of mental infection. Above a hundred unmarried women were seen roving about in consecrated and unconsecrated places, and of course the consequences were soon perceptible. Gangs of idle vagabonds, also, imitating the convulsions of the possessed, spread the disgusting spasmodic disease like a plague; and roving from city to city, provoked scenes as strange as they were detestable. For in maladies of this kind, the susceptible are infected as easily by the appearance as by the reality.

Another variety of the "Dancing Plague" visited the town of Strasburg in the year 1418. This was called the "St. Vitus's Dance," and must have been somewhat similar to the chorea of the present day. Then, however, it was epidemic, and produced the same infatuation among the people as the St. John's Dance in the towns of Belgium and the Lower Rhine. Those who from curiosity followed the swarms of dancers were visibly affected by the sight, and evinced more or less confusion and absurdity in their behaviour. Speedily, unable to resist the frenzy, they joined in the mad revels, and passed days and nights in the streets, capering to the sound of the bagpipes and other instruments. Strange it must have been to have seen hundreds of men and women dancing and jumping in the public market-places, the streets and lanes, and continuing the exertion until completely exhausted. Over stools, forms, and tables, sometimes purposely put in their way, even women far advanced in the family-way would dance until they could stir neither hand or foot. The consequences of this mental plague were a fearful loss of life, and the visitation of thousands with incurable aberration of mind, and disgusting distortions of body.

It would appear that this dancing mania was a phenomenon well known in the middle ages, previous to its great accession in 1374. Thus, in the year 1237, upwards of a hundred children are said to have been suddenly seized with this disease at Erfurt, and to have proceeded, dancing and jumping, along the road to Arnstadt. When they arrived at that place, they fell exhausted to the ground, and, according to an old chronicle, many of them, after they were taken home, died, and the rest remained affected to the end of their lives with a permanent tremor. Another occurrence was related to have taken place on the bridge at Utrecht, on the 17th day of June, 1278, when two hundred fanatics began to dance, and would not desist until a priest passed, who was carrying the host to a person that was sick, upon which, as if in punishment of their crime, the bridge gave way and they were all drowned.

If we endeavour to fathom the causes, the *fons et origo* of this strange malady, we shall find that these were deeply laid in the misery and physical sufferings of the people. We shall comprehend how despair sought relief in the intoxication of an artificial delirium. At the anniversary of St. John, bones and other rubbish were heaped together to be consumed in smoke, while persons of all ages danced round the flames as if they had been possessed, in the same way as at the Palilia, an ancient Roman lustration by fire, whereat those who took part in it sprang through a fire made of straw. "There is good ground for supposing," says Hecker, "that the frantic celebration of the festival of St. John, A.D. 1374, only served to bring to a crisis a malady which had been long impending; and if we would further inquire how a hitherto harmless usage which, like many others, had but served to keep up superstition, could degenerate into so serious a disease, we must take into account the unusual excitement of men's minds, and the consequences of wretchedness and want. The bowels, which in many were debilitated by hunger and bad food, were precisely the parts which in most cases were attacked with excruciating pain; and the tympanitic state of the intestines, points out to the intelligent physician an origin of the disorder which is well worth consideration."

That the St. John's and St. Vitus's dance was propagated or communicated by sympathy, there can be no doubt. The great medical reformer, Paracelsus, was clearly of this opinion. Sensual impressions, he says, find their way to the heart—the seat of joys and emotions; they overpower the opposition of reason; and whilst "all other qualities and natures" are subdued, incessantly impel the patient, in consequence of his original compliance and his all-conquering imagination to imitate what he has seen.

It may naturally be imagined, that occasionally the frenzy was feigned from interested motives. Doubtless. But the evidence is all powerful as to the absence generally of all motives for imposture, and the too sad reality of the infliction. The severest punishments of the magistrate were useless. Those possessed often continued to dance without intermission, until their very last breath was expended. Their fury and extravagance of demeanour so completely deprived them of their senses, that many of them dashed their brains out against the walls and corners of buildings, or rushed headlong into rapid rivers, where they found a watery grave. Roaring and foaming as they went, the bystanders could only succeed in restraining them by placing benches and chairs in their way, that, by the high leaps they were thus tempted to take, their strength might be sooner exhausted. Music was the only thing that had any effect upon the transports, although the paroxysms were as often brought on and increased, as mitigated by it. On account of its influence the magistrates hired musicians for the purpose of carrying the St. Vitus's dancers so much the quicker through the attacks, and directed that athletic men should be sent among them in order to complete the exhaustion, which had been often observed to produce a good effect. One instance of the persistence of the frenzy is so curious, that it deserves repetition. It is related by Felix Platir, that he remembered in his youth the authorities of Basle having commissioned several powerful men to dance with a girl who had the dancing mania, till she recovered from the disorder. They successively relieved each other, and this singular mode of cure lasted above four weeks, when the patient fell down exhausted, and being quite unable to stand, was carried to an hospital, where she recovered. She had remained in her clothes all the time, and entirely regardless of the pain of her lacerated feet, she had merely sat down occasionally to take some nourishment or to slumber, during which the hopping movement of her body continued.

Nor were the mischievous propensities of the dancers always confined to themselves; they were not always solely their own enemies. The peaceable inhabitants were prohibited wearing red garments, because, at the sight of this colour, those affected became so furious that they flew at the persons who wore them, and tried to injure them. The more opulent, therefore, employed confidential attendants to accompany the maniacs to see they did no mischief.

We cannot pass over this period of history without alluding to *lycanthropy* as a true mental epidemic. The delusion spread in proportion to the efforts made to suppress it. Lycanthropy was an extraordinary species of insanity, depending doubtless upon a morbid condition of the body, as it was observed that the maniacs were hollow-eyed, pale, and forlorn; their tongues were dry, and they were troubled with immoderate thirst. These poor souls fancied themselves metamorphosed into wolves, and therefore lay hid all day, and at night went abroad, howling and barking, and tearing open dead men's graves. This disease originally existed in the province of Arcadia, in Greece, a country abounding in forests, morasses, and pasture lands; and in process of time spread over Europe, afflicting not only the Roman, but the German and Sarmatian nations. This hallucination is now, happily, banished from the earth, if we except the disorder which is said to occur among the aborigines of Brazil. After the Indian has wandered about for a time, pale, silent, reserved, with a confused, fixed stare, he suddenly breaks loose in the evening after sunset, runs raving through the village, howls, turns up the graves, and rushes into the woods.*

Turn we now to another and an opposite part of Europe, and we shall observe that, coeval with the outbreak of the St. John's dance in Germany, a malady having a close similitude to it, and equally extraordinary in all its phenomena, made its appearance in Italy. This affords a most instructive subject for contemplation, as the simultaneous visitation of a

* Feuchstersleben, p. 253.

moral pestilence in distant countries leads us to infer some common though mysterious cause. The disease was called "tarantism," from the belief that it arose from the bite of the tarantula, a ground spider common to certain parts of Italy. For some centuries tarantism prevailed as a great epidemic, having originated in Apulia and from thence spread over the whole peninsula. Isolated cases of this malady are still occasionally met with, and the dance called the "Tarantella" must be familiar to every one who has visited the land of the Cæsars. Hecker, after enumerating the disasters which ravaged Italy even more than other countries, gives the following lucid explanation of the origin of tarantism:—

"Men's minds were everywhere morbidly sensitive; and as it happens with individuals whose senses, when they are suffering under anxiety, become more irritable, so that trifles are magnified into objects of great alarm, and slight shocks, which would scarcely affect the spirits when in health, give rise in them to severe diseases, so was it with this whole nation, at all times so alive to emotions and at that period so sorely pressed with the horrors of death. The bite of venomous spiders, or rather the unreasonable fear of its consequences, excited at such a juncture, though it could not have done so at an earlier period, a violent nervous disorder, which, like St. Vitus's dance in Germany, spread by sympathy, increasing in severity as it took a wider range, and still further extending its ravages from its long continuance. Thus, from the middle of the fourteenth century, the furies of *the dance* brandished their scourge over afflicted mortals; and music, for which the inhabitants of Italy, now probably for the first time, manifested susceptibility and talent, became capable of exciting ecstatic attacks in those afflicted, and then furnished the magical means of exorcising their melancholy." But we must proceed with a description of the malady.

Tarantism was a true nervous disorder, tending to show the great influence of the imagination in the production of disease. Although females, from their more excitable nature, were excessively prone to the infection, yet the strongest men of all classes in society were subjected to its power. Nor was the complaint confined to the natives of Italy alone. Foreigners of every colour and of every race, negroes, gipsies, Spaniards, Albanians, were in like manner affected by it. Neither youth nor age afforded protection against the effects of the bite or the sight of the sufferers; so that even old men of ninety threw aside their crutches at the sound of the tarantella, and as if some magic potion, restorative of youth and vigour, were flowing through their veins, joined the most extravagant dancers. It is said that even deaf people were not exempted, catching the disorder through the eye.

The whole mania of tarantism may be said to have been founded in the belief, which the strongest and healthiest could not withstand, that the bite of the ground spider was certain death. If those who were bitten escaped with their lives, they were seen pining away in a desponding state of lassitude. They felt they were doomed, and therefore gave way to hopeless melancholy. The mental malady was progressive in its nature. Unaccountable emotions, strange desires and morbid sensual irritations, ushered in the attack. Then followed loss of voice, occasional blindness, vertigo, and sleeplessness. The body, sympathizing with the mind, became weak and disordered. The digestive apparatus especially suffered. Finally, complete insanity supervened, and the poor sufferers either ended their miserable existence by suicide, or prolonged it away from the haunts of men among the tombs of the dead.

The Tarantati, it appears, had their fancies and antipathies, but in a much more marked manner than their phlegmatic brother dancers of Germany. Their excitement was great at the view of anything with metallic lustre.

This suggests a comparison between those bitten by a mad dog in this country and those stung by the tarantula in Italy. But here the sight of water generally increases the patient's sufferings. The spider-bitten, on the contrary, had a most ardent longing for the limpid stream. The greatest pleasure was afforded them by the sight of clear water. They held glasses of it in their hands while dancing, and took every opportunity of bathing their arms and heads in the pure element. Their love for the sea was most extraordinary. The boundless expanse of the blue ocean had irresistible attractions, and they lost themselves in its contemplation. Some in whom this susceptibility was carried to its highest pitch, cast themselves into the waves with blind fury, in a transport of pleasurable excitement.

Nor should we omit making allusion to the agreeable sensations produced by certain colours. Unlike the dancers of St. John who detested red colours, these poor lunatics delighted in it, and carried about a red handkerchief for their gratification. Some, it appears, preferred yellow, others black, while others were enraptured with green; but whatever was the tint chosen, their rage for it was most extraordinary. At the present day we can hardly suppose it possible that such a scene could have occurred as the following. "No sooner did the patients obtain a sight of the favourite colours than, new as the impression was, they rushed like infuriated animals towards the object, devoured it with their eager looks, kissed and caressed it in every possible way; and gradually resigning themselves to softer sensations, adopted the languishing expression of enamoured lovers, and embraced the handkerchief, or whatever other article it might be which was presented to them, with the most intense ardour while the tears streamed from their eyes, as if they were completely overwhelmed by the inebriating impression on their senses. Their rage was equally excited by colours which they disliked.

On this strange malady it was found that nothing had the slightest effect but music, and this only of a particular kind. Hence tarantellas were composed expressly for the purpose. However hopeless the cases might have appeared, the patients, tortured with pain or collapsed from exhaustion, at the first note of their favourite melodies sprang from their couches with new life and spirit, and danced for hours together without apparent fatigue, until, covered with a kindly perspiration, they were sensibly relieved from their sufferings. If the music stopped but for a moment before they were exhausted, all the previous symptoms returned, and they relapsed into a deeper state of dejection and oppression. One thing very strange was noticed at these exhibitions. The most uneducated boors moved to the tunes with grace and elegance, and seemed suddenly to have acquired, as if by inspiration, a refined musical ear, detecting every false note, although they had previously never in their lives manifested any perception of the enchanting powers of harmony.

It is difficult to determine, at this distant day, how far deception and fraud may have been mixed up with tarantism. Cases of imposture must have occasionally occurred. But when we consider the real bodily sufferings the dancers endured, and the cruelty with which they were generally treated by the spectators, we cannot reasonably doubt the genuineness of the malady. Tarantism was a disease of the imagination, fostered and propagated by sympathy and imitation. A true mental epidemic. It may be very much doubted whether a remedy could be found, even in these days of enlightenment, were we visited by such a dancing plague. It is difficult to minister to a mind diseased. But music, although undoubtedly serviceable in bringing on a crisis in those bitten by the mania, must have greatly aided the spread of the complaint. Andral, in noticing these epidemic dances of the middle ages, says they reminded him of the experiments of Fleurens on the section of the peduncles of the cerebellum in pigeons and rabbits, which immediately after the operation commenced to move in circles or in a retrograde direction until completely exhausted. Many of the symptoms, especially the tympanic state of the bowels, suggest the idea of hysteria. But independently of the fact that on the whole more males than females were troubled with tarantism, there is abundant evidence to prove that, although frequently complicated with that proteiform malady, it yet ran its own course uninfluenced by it.

That hysteria is itself a distinct contagious nervous affection, every medical practitioner can bear testimony. Its epidemic nature in hospitals is well known. Zimmerman, in his work on "Solitude," tells us that in a large convent in France a nun one day began to mew like a cat. Shortly afterwards other nuns did so. At last all the nuns mewed together every day, at a certain time, for several hours together, to the great scandal of the neighbourhood. There was also in Germany a still more remarkable convent epidemic, described by Cardan. A nun in a German nunnery fell to biting her companions. In the course of a short time all the fair sisters fought together with their teeth. The news of this infatuation among the nuns soon spread, and it now passed from convent to convent throughout a great part of Germany, principally Saxony and Brandenberg. It afterwards visited the nunneries of Holland, and at last the nuns had the biting mania even as far as Rome.

Following the course of history, we find that in the fifteenth and sixteenth centuries other errors had taken the place of those exploded. This was the epoch of magic and belief in compacts with the devil—the time of magicians, sorcerers, witches, and demonomaniacs. These were real delusions of the mind, and were propagated like other contagious affections. The thousands of poor wretches who expiated in flames and torments the misfortune of having lost their reason, served but to spread the epidemic malady. The confessions wrung from the victims were sincere. They thoroughly believed they had communications with the Evil One, and even held intercourse with him of a sexual character.

Martin Luther himself could not escape from the influences of his age. He fought with the devil, seized him by the horns and overthrew him. At another time, the demon conquered in his turn, and the reformer came out worsted from the combat. It is well known that, having failed once to convince his Satanic majesty by his arguments, he threw the ink-bottle at his head.

A species of mental epidemic was predominant at the beginning of the eighteenth century, in various parts of Hungary, Moravia, Silesia, and Lorraine. It was called Vampirism. Those who laboured under the malady, believed that the souls of their enemies after death would appear to them in various disguises, and even injure them and their cattle, unless their bodies were burnt. Many dreamt that these malicious spectres took them by the throat, strangled them, and sucked their blood. Others swore they saw these cruel goblins do so, and thus the malady became general. The terror occasioned by these visions was ordinarily so great, that after having experienced it two or three times, the person was exhausted, and died in a state of syncope. The evil was carried to that extent that, not being able to check these flights of imagination, the magistrates were obliged to allow the sanctuaries of the dead to be violated in order to save the living.

Passing over the numerous instances of epidemic disorders of the mind occurring in factories and other crowded and ill-ventilated assemblies, from causes altogether insignificant, we come to those occasioned by the excitement of the feelings during the act of devotion. Religion is of all other enthusiastic infatuations the most fertile in mental derangement, and the infection spreads with the greatest facility by sympathy. No otherwise can we account for the frenzied actions of the followers of Mahomet and other Eastern propagandists. The history of our own church furnishes innumerable proofs. None more prominent among these than the eccentricities of the *Convulsionnaires* in France, in the year 1731. The origin was thus. Those who visited the tomb of the Deacon Paris, in the cemetery of St. Medard, spread a rumour that miracles took place there. Votaries were seized with convulsions and tetanic spasms, rolled upon the ground like persons possessed, had violent contortions of their head and limbs, and suffered the greatest oppression, accompanied by quickness and irregularity of pulse. This novel occurrence excited the greatest sensation all over Paris, and an immense crowd of people resorted daily to the cemetery, in order to see so wonderful a spectacle. The disorder soon increased, until it produced in nervous women *clairvoyance*—a phenomenon till then unknown. One female especially attracted attention, who, blindfold, and, as it was believed, by means of the sense of smell, read every writing that was placed before her, and distinguished the characters of unknown persons. The very earth taken from the grave of the deacon was soon thought to possess miraculous power. It was sent to numerous sick persons at a distance, whereby they were said to have been cured; and thus this nervous disorder spread far beyond the limits of the capital. At one time, it was computed that there were more than eight hundred decided *Convulsionnaires.* The disorder itself assumed various forms, and augmented by its attacks the general excitement.

Many persons, besides suffering from the convulsions, experienced violent pain, which required the assistance of their brethren of the faith. On this account they, as well as those who afforded them aid, were called by the common title of *Secourists.* The modes of relief adopted were remarkably in accordance with those which were administered to the St. John's dancers and the tarantati, and they were in general very rough; for the sufferers were beaten and goaded in various parts of the body with stones, hammers, swords, clubs, &c.; of which treatment the defenders of this extraordinary sect relate the most astonishing examples, in proof that severe pain is imperatively demanded by nature in this disorder, as an effectual counter-irritant. The Secourists used wooden clubs, in the same manner as paviors use their mallets; and it is stated that some Convulsionnaires have borne daily from six to eight thousand blows thus inflicted, without danger. Sometimes, however, the patients died from the effects of the *Grand Secours.* One Secourist administered to a young woman who was suffering under spasm of the stomach the most violent blows on that part. Sometimes the patients bounded from the ground, impelled by the convulsions, like fish when out of water; and this was so frequently practised at a late period, that the women and girls, when they expected such violent contortions, not wishing to appear indecent, put on gowns made like sacks closed at the feet. The female sex especially was distinguished by all kinds of leaping, and almost inconceivable contortions of body. Some spun round on their feet with incredible rapidity, as is related of the dervishes; others ran their heads against walls, or curved their bodies like rope-dancers so that their heels touched their shoulders. All this degenerated at length into decided insanity. The most absurd practices were resorted to by the fanatics. Some had a board placed across their bodies, upon which a whole row of men stood; and as, in this unnatural state of mind, a kind of pleasure is derived from excruciating pain, some too were seen who caused their bosoms to be pinched with tongs; while others, with gowns closed at the feet, stood upon their heads, and remained in that position longer than would have been possible had they been in health. Pinault, the advocate, who belonged to this sect, barked like a dog some hours every day; and even this found imitation among the believers.

That sect of English Methodists called the *Jumpers,* surpass, if possible, the French *Convulsionnaires.* It is difficult in their case to draw the line between religious ecstacy and a perfect disorder of the nerves. By the use of certain unmeaning words they work themselves up into a state of religious frenzy, in which they seem to have scarcely any control over their senses. They then begin to jump, with strange gestures, repeating this exercise with all their might until they are exhausted; so that it not unfrequently happens that women, who, like the Mænades, practise these religious exercises, are carried away from the midst of them in a state of syncope, whilst the remaining members of the congregation, for miles together on their way home terrify those whom they meet by the sight of such demoniacal ravings. There are never more than a few ecstatics, who by their example excite the rest to jump, and these are followed by the greatest part of the meeting, so that these assemblages of the Jumpers resemble for hours together the wildest orgies, rather than congregations met for Christian edification. Most of our readers will remember the strange scenes exhibited in the chapel of the late Mr. Irving. It is hardly too much to affirm that the reverend gentleman himself and nearly all his followers were the subjects of monomania of a contagious nature, and this without throwing the slightest shadow of suspicion on their sincerity.

Mrs. Trollope, in her "Domestic Manners of the Americans," and other writers, give us ample details of the same kind of aberration, but carried to a still greater pitch of absurdity, in the New World. At the camp meetings, at which thousands assemble for divine worship, every folly and absurdity is committed calculated to bring their religious faith into disrepute. Hundreds have swooned away, worn out by raving and jumping. In the state of ecstacy into which they are thrown, women have publicly stripped themselves and plunged into the rivers, whilst others have miscarried during the fits of convulsion. A sect called the Barkers manifest symptoms of complete aberration of intellect. Whole bands are seen moving on all fours, barking and growling, and otherwise indicating the degradation into which they have fallen. This mad infatuation is readily communicated by sympathy to the bystanders, especially those of a tender age or nervous constitution.

We think that, in the investigation of the causes of mental epidemics, we should always bear in mind the influence of internal organic changes on the intellectual disorder. The opinion of M. Broussais is valuable on this subject. He says:—"A point well worthy of our notice is the frequent manifestation, through the deranged perceptions of the insane, of the seat of the abdominal lesion existing simultaneously with the mental derangement. Thus some are tormented by a dread of poison; others, by a strange and bizarre hallucination, believe in the existence within them of some extraordinary creature, some savage animal, perpetually gnawing, rending, and biting their intestines. All these sensations exist specially in those whose intestines are affected. It is a real feeling badly interpreted; but it is also a valuable guide in the treatment of the disease."

What better illustration of this can we desire than the anecdote narrated by Andral in one of his lectures? He calls it the history of the epidemic monomania of St. Souard, and never was there a history more curious and extraordinary. A battalion of French soldiers, during the toils and dangers of a campaign, were marching on a certain point on a hot and overcoming day, and at double the usual speed. Their strength was 800, all hardy, seasoned, and courageous men, careless of danger, despising the devil, and little occupied with thoughts of ghosts and phantasmagoria. On the night of the occurrence in question, the battalion was forced to occupy a narrow and low building, barely calculated to accommodate 300 persons. Nevertheless they slept, but at midnight one and all were roused by frightful screams issuing from all quarters of the house, and to the eyes of the astonished, affrighted soldiers, appeared the vision of a huge dog, which bounded in through the window, and rushed with extraordinary heaviness and speed over the breasts of the spectators. The soldiers quitted the building in terror. Next night, by the solicitations of the surgeon and chef-de-battaillon, who accompanied them, they again resumed their previous quarters. "We saw," says the narrator, "that they slept; wide awake we watched the arrival of the hour of the preceding panic, and midnight had scarcely struck when the veteran soldiers for the second time started to their feet. Again they had heard the supernatural voices; again the visionary hound bestrode them to suffocation. The chef-de-battaillon and myself heard or saw nothing of these events." Here, gentlemen, proceeds Andral, is a curious fact, and it is perhaps more worthy of attention, as it seems to point out the especial operation of physical causes in the production of monomania, and in the direction of the delusion towards the organs, namely those of respiration, which had chiefly suffered in the previous marches, and in the suffocating atmosphere of the den in which they slept.

From this epitome of most voluminous evidence we have here brought forward, we believe it may be considered established that there are certain species of insanity of a contagious nature, and of a temporary duration, consisting of a suspension of the healthy action of the intellectual functions: that the infection is capable of being spread as an epidemic through the media of example, sympathy, and imitation.

Nor should it be supposed that these strange details marked only the errors and darkness of by-gone times. We have only to look around us to observe, that such indications of human weakness are of daily occurrence among the most civilized nations of the earth. In this country the commission of a great and extraordinary crime produces, not unfrequently, a kind of mania of imitation in the district in which it happens. In this way we account for the infatuation of several weak persons shooting at the Queen in succession, and when a foolish girl threw herself from the Monument, others were found silly enough to follow her example.

In a neighbouring state the same spirit of mischievous sympathy has been repeatedly evinced. Thus, a supposed miracle having been performed before the gate of the convent of St. Geneviève, such a number of similar occurrences happened on the same spot in a few days, that the police were compelled to post a peremptory notice on the gate, "prohibiting any individuals from working miracles on the place in question." A few years since, at the Hôtel des Invalides, a veteran hung himself on the threshold of one of the doors of a corridor. No suicide had occurred in the establishment for two years previously; but in the succeeding fortnight five invalids hung themselves on the same cross-bar, and the governor was obliged to shut up the passage. Here is a case exactly similar to the London Monument-mania. During the last days of the empire an individual ascended the column in the Place Vendôme, and threw himself off, and was dashed to pieces. The event excited a great sensation. In the course of the ensuing week four persons imitated his example, and the police were obliged to proscribe the entrance to the column.

Instances of a similar kind could be cited *ad infinitum*, but why should we look for them when we ourselves are witnesses of mental disturbance on a scale of fearful magnitude? The demon of revolution is a veritable mania, that is now stalking with giant strides over the peace of Europe. A dreadful epidemic, spreading by sympathy and imitation around the focus from whence it started, and involving in its toils, subduing by its pestilential breath, all those whom previous physical suffering or mental incapacity had predisposed for victims. Onwards it will tend, creating horror and dismay among the *uninfected,* until, like the dancing plague, it has worn itself out by its vagaries, or is checked by the united good sense of the community. But who can tell what horrors may ensue ere this takes place? It is useless for those with whom the storm has originated, to attempt to stop its progress. Downwards it will thunder, crushing everything that impedes its progress. The floodgates once removed, all must go with the stream or be overwhelmed in the roaring cataract.

There is such a thing as murder-madness, which springs up insensibly. Those who are cursed with this mania desire to spill blood—nay, more, to drink it, to slake their thirst with it with all the carnivorous longing of tigers. Horrible to think too, this atrocious taste spreads by imitation among multitudes of people, becomes an epidemic murder-madness, only ceasing when it has no more victims to immolate. Ancient Rome and every other city of revolutions has chapters of this character in its history, and such was the origin of the carnage of the Septembrizers in the first French revolution. Absolutely drunk with blood, they persevered in their butcheries for days together. At this present day the slightest spark may fire a magazine of equal terror.

Many circumstances tend to show that this political crisis was the result of impulse much more than reason. Doubtless the first leaders were of sound, and perhaps strong minds, and some cause existed for the revolutionary movement; but as soon as the feelings of the people were excited these men sank into insignificance. The accidental firing of a gun set all in commotion, and immediately innumerable maniacs, led on by the morbid principle of imitation, were to be found among the crowd. Fortunately the delirium was allowed to subside partially from being unopposed, or it would most probably have hurried its victims into the most fearful excesses. Still a sufficient number of acts of madness were committed to show that the poison was working. Great must have been the genius of that man who was able to "ride on the whirlwind and direct the storm,"—who was able with his eye to control the raging mass around him, and, like another Orpheus, tame by his persuasive tongue the wild hyenas at his feet. The man, Lagrange, who presented a pistol at his head, and was immediately afterwards carried away raving mad to an asylum, was, we are assured, but an ordinary specimen of the political maniac.

Another and a strong proof that the revolutionary spirit is a moral epidemic is, that it is taking the same course in all essential points as it did on a former occasion. The instinct of imitation completely baffles and overpowers reason. So fully are we impressed with the truth of this observation, that we would venture to predict that the whole will be the same scene acted over again, not with the same rapidity as before, because the lunacy is not at the same height, but with the same certainty. No other mode of cure presenting itself to the mind, it may even be a matter of question whether, as was practised by the authorities of Italy and Germany, it would not be better to administer suitable *stimulants* in order to hasten the crisis, provided always some guardian angel could be present to see that the maniacs neither injured themselves or others. If we are to judge by the experience of the past, to form our opinion from the annals of the large institutions devoted to the insane, we can readily determine the specific nature of the ailments that will have to be treated for the next few years. Political revolutions and republican governments are ever most fruitful in mental aberration, so that there will be numberless cases of violent mania from extraordinary excitement, ambitious monomania from dreams of impossible wealth and glory, and hopeless melancholy from disappointed ambition and ruined plans and prospects.

There are certain grave reflections that will naturally suggest themselves to those who have the care of the alienated, in considering the epidemic nature of certain mental states and affections. They will see the necessity of repressing the very slightest eccentricities of manner or conduct among their patients, and of carefully separating from the rest those who exhibit an infectious nervous disorder. Their own conduct must be especially steady and sedate, without infringing on that cheerfulness or even gaiety of disposition calculated to infuse hope and confidence in the afflicted. Finally they would do well to guard all persons of a nervous or excitable temperament, from seeing or more particularly from associating with the insane. In these days, when the humane and enlightened system of management is carried to its furthest limits, this caution is not needless. Constant association with lunatics may prove, to a certain class of minds, extremely dangerous. Sympathy and imitation are beyond the control of the will, and are not to be combated by reason. In the histories previously detailed we have seen that healthy and powerful men have been drawn into the vortex of epidemic mania, and we have ourselves known several instances of persons whose minds have become impaired, from being constantly associated with lunatics. All those, therefore, who are brought in contact or personal communication with the insane, should exercise a vigilant and careful control over their own thoughts, feelings, and conduct; and those who from hereditary taint or mobile temperament are predisposed to diseased mental emotion should avoid their society altogether. This remark of course applies more particularly to those whose sympathetic nature and peculiarity of organic structure peculiarly dispose them to every variety of derangement of the nervous system.

Since the above remarks were written, we have perused an abstract of a paper on the effect of political emotion, communicated to the *Académie de Medicine,* by M. Belhomme. It appears that in 1830, M. Belhomme communicated to the Société Medico-Pratique several cases of insanity brought to his establishment, in which the exciting cause had been the events of July. In 1832, some new cases, reported by M. Belhomme to the same society, went to prove that the *émeutes,* which disturbed Paris in 1831 and 1832, had given rise to a great number of acute alienations. In 1848, we have a fresh report of ten cases, which show that the fright caused by the state commotions has given rise to several attacks of mental derangement. After having presented the preceding facts in detail to the Academy (whose members seemed extremely interested in the paper), M. Belhomme concluded by the following reflections:

All the patients who have come under his notice were very strongly predisposed to insanity; they had either had previous fits of mania, or were born of insane parents, or else were remarkable for eccentricities and irrational habits. The cases were mostly acute, went rapidly through their phases, and had a favourable termination. Of the above-named ten patients, eight were cured, one was declared incurable, and the last died. The treatment was of a sedative character. In twenty such cases, which were observed at different periods, the affection presented the same symptoms, progress, and termination.

The author thinks, on the whole, that the political events which have successively shaken the social system in France for the last fifty years, have notably increased the number of the insane, and he quotes Esquirol and Pariset, who entertained the same opinion. Pariset, in particular, acknowledges that any considerable and rapid change, either in the physical or moral state of things, is pernicious both to health and intelligence.

We subjoin M. Belhomme's conclusions:—

1. One of the moral causes which act on the development of mental derangement is the perturbation resulting from revolutions.

2. That insanity, in such cases, singles out predisposed individuals, who are, in some degree, on the brink of an attack.

3. That it assumes an acute form, and is therefore more susceptible of cure than otherwise.

4. That the sedative treatment is the best, particularly long-continued warm baths, with cold affusions on the head.

5. That derivatives towards the intestinal canal and the skin generally bring the disease to a favourable termination.

6. And, lastly, that a well-regulated *moral* treatment powerfully aids the cure.

